# An in-depth evaluation of metagenomic classifiers for soil microbiomes

**DOI:** 10.1186/s40793-024-00561-w

**Published:** 2024-03-28

**Authors:** Niranjana Rose Edwin, Amy Heather Fitzpatrick, Fiona Brennan, Florence Abram, Orla O’Sullivan

**Affiliations:** 1 Teagasc, Moorepark Food Research Centre, Moorepark, Fermoy, Cork, Ireland; 2 University College Dublin, Belfield, Dublin 4, Ireland; 3 Teagasc, Soils, Environment and Landuse Department, Johnstown Castle, Wexford, Ireland; 4 Functional Environmental Microbiology, School of Biological and Chemical Sciences, Ryan Institute, University of Galway, Galway, Ireland; 5 VistaMilk SFI Research Centre, Cork, Ireland

**Keywords:** Shotgun metagenomics, Taxonomic classifier comparison, Soil microbiome, Genome taxonomy database, Kraken2, Bracken, Kaiju, MetaPhlAn

## Abstract

**Background:**

Recent endeavours in metagenomics, exemplified by projects such as the human microbiome project and TARA Oceans, have illuminated the complexities of microbial biomes. A robust bioinformatic pipeline and meticulous evaluation of their methodology have contributed to the success of these projects. The soil environment, however, with its unique challenges, requires a specialized methodological exploration to maximize microbial insights. A notable limitation in soil microbiome studies is the dearth of soil-specific reference databases available to classifiers that emulate the complexity of soil communities. There is also a lack of in-vitro mock communities derived from soil strains that can be assessed for taxonomic classification accuracy.

**Results:**

In this study, we generated a custom in-silico mock community containing microbial genomes commonly observed in the soil microbiome. Using this mock community, we simulated shotgun sequencing data to evaluate the performance of three leading metagenomic classifiers: Kraken2 (supplemented with Bracken, using a custom database derived from GTDB-TK genomes along with its own default database), Kaiju, and MetaPhlAn, utilizing their respective default databases for a robust analysis. Our results highlight the importance of optimizing taxonomic classification parameters, database selection, as well as analysing trimmed reads and contigs. Our study showed that classifiers tailored to the specific taxa present in our samples led to fewer errors compared to broader databases including microbial eukaryotes, protozoa, or human genomes, highlighting the effectiveness of targeted taxonomic classification. Notably, an optimal classifier performance was achieved when applying a relative abundance threshold of 0.001% or 0.005%. The Kraken2 supplemented with bracken, with a custom database demonstrated superior precision, sensitivity, F1 score, and overall sequence classification. Using a custom database, this classifier classified 99% of in-silico reads and 58% of real-world soil shotgun reads, with the latter identifying previously overlooked phyla using a custom database.

**Conclusion:**

This study underscores the potential advantages of in-silico methodological optimization in metagenomic analyses, especially when deciphering the complexities of soil microbiomes. We demonstrate that the choice of classifier and database significantly impacts microbial taxonomic profiling. Our findings suggest that employing Kraken2 with Bracken, coupled with a custom database of GTDB-TK genomes and fungal genomes at a relative abundance threshold of 0.001% provides optimal accuracy in soil shotgun metagenome analysis.

**Supplementary Information:**

The online version contains supplementary material available at 10.1186/s40793-024-00561-w.

## Background

Shotgun metagenomics is defined as untargeted sequencing of all the genomes present in a sample [1] facilitating a de novo analysis of both taxonomical and functional profiles without any prior knowledge [2]. This approach has not only significantly expanded the tree of life by incorporating genomes from previously unculturable microbial lineages but is also poised to gain prominence due to the falling costs and heightened efficiency and speed of sequencing technologies [3, 4]. In the field of antimicrobial resistance, the soil microbiome is emerging as one of the critical frontiers. Studies suggest that a large number of new antimicrobial resistance genes may be contained in soil ecosystems [5–10]. In addition, soil plays an integral role in a host of essential functions, such as nutrient cycling, carbon sequestration, plant pathogen resistance, drought tolerance, amongst others. It is imperative that the wet-lab and bioinformatic methods applied permit us to accurately investigate the complexity and potential of the soil microbiome.

Several taxonomic classifiers have been developed for analysing taxonomic abundance from the growing volume of sequenced data [11–17]. A considerable number of these previous studies have featured Kraken2, Kaiju, and MetaPhlAn in their comparative analysis of metagenomic classifiers [11–19]. There is, however, a gap in benchmarking efforts targeting shotgun sequencing in the context of soil microbiomes, as most previous studies have focused on 16S rRNA amplicon sequencing, or long-read sequencing focused on food microbiomes or clinical pathogens. These studies scrutinize a variety of parameters including sequencing platform, classifier choice, sequencing depth, relative abundance threshold or filtering thresholds (which refer to the set criteria for determining the minimum abundance a microbial entity must have in a sample to be included in the data analysis), and the effectiveness of using trimmed versus assembled contigs in the analysis [20]. Nonetheless, the intricate landscape of soil datasets, from the presence of a significant number of uncultured microbes (leading to an incomplete reference database) to an inherently elevated microbial complexity, still lacks a focused benchmarking study.

A significant hurdle in adequately emulating such a complex microbiome in sequenced data lies in the formulation of a mock community that is reflective of the vast diversity of microbial strains associated with soil. It is important to note that our inability to culture many soil-identified microbes in laboratories results in a reliance on mock communities that may poorly represent the true microbial diversity in soil samples. Despite recent efforts to develop appropriate mock communities, (including the most extensive soil bacterial strain mock community to date containing 254 strains [21]), the sheer diversity found in real soil samples is far greater than can feasibly be incorporated. Studies report up to 10^4^ microbial species are found per gram of soil [22] and there are 888 bacteria, 24 archaea, and 6 fungal strains documented in the public database, RefSoil [23]. This significant disparity underscores the current limitations in creating a mock community that mirrors the diversity and complexity seen in natural soil microbiomes. This study sets a new benchmark representing the soil microbiome with a total of 2795 unique strains, including 2621 bacteria, 60 archaea, and 114 fungal strains.

In typical soil shotgun studies, soil taxonomy serves a pivotal role in correlating functional analysis elements such as biosynthetic gene clusters, the antimicrobial resistance profile, or nutrient cycling genes. However, the study by Laura et al. 2022, takes a step forward by leveraging the classifier Kraken2 for a nuanced classification of identified antimicrobial resistance genes in soil samples, potentially offering a more refined insight into microbial community dynamics and functionalities [24]. Despite this method's potential for enhanced precision and depth, benchmarking studies are vital to confirm these initial findings. Given the current limitations, comprehensive in-silico studies have emerged as a vital tool to enhance the accuracy and depth of soil microbiome analyses. Furthermore, in-silico approaches allow for the efficient analysis of highly diverse sample types, enhancing overall statistical effect size while minimizing associated costs. These methods are not only seamless in their application but are highly adaptable to technological advancements thereby allowing reanalysis of data. Moreover, these methods also negate the influences of sequencing platform discrepancies and manual errors commonly encountered in wet-lab method benchmarking [25].

In this study, we employ both simulated and real-world soil data to ascertain the misclassification rates at species, genus and family levels when utilising commonly adopted classification tools used in Illumina shotgun metagenomic analysis. We first created a soil-specific database that compiled genomes of soil bacteria, archaea, and fungi from public databases. Leveraging this in-silico representative soil dataset, we performed a comparative analysis of three prominent taxonomic classifiers: Kraken2 (supplemented with Bracken), Kaiju, and MetaPhlAn (versions 3 and 4). The objective of this study was to evaluate the ability of each classifier to precisely detect and quantify the archaeal, bacterial, and fungal communities in our in-silico samples and to understand the elements driving this precision. Having determined the most efficient classifier and parameter setup, we applied this refined approach to analyse real soil data, enabling a thorough comparison with the conclusions derived from prior studies.

## Material and methods

Our research aimed to identify the optimal taxonomic classifier and parameters for taxonomic profiling of shotgun metagenomic data from soil. Notably, a comprehensive public soil-specific genome database encompassing both culturable and non-culturable soil genome information is currently unavailable. To address this gap, we developed our database, merging genomes from the NCBI database with those from the RefSoil database, which exclusively houses genome information of culturable soil-specific microbes. This resulted in an in-depth collection of 2795 unique strains, encompassing bacteria, archaea, and fungi. Leveraging this soil-specific in-silico mock community, we simulated ten NovaSeq runs, each comprising 20 samples with 150 bp read length. After thorough quality checks and trimming, we assembled the reads into contigs. Both the refined reads and assembled contigs were then subject to analysis by various taxonomic classifiers, including Kaiju, MetaPhlAn 3, MetaPhlAn 4, Kraken2 using a default database referred as “Kraken2 (default)” and a modified Kraken2 version utilizing a custom database from GTDB-TK genomes, which we will refer to as "Kraken2 (custom)" henceforth. The core of our analysis was to evaluate the capacity of each classifier in precisely detecting and quantifying the archaeal, bacterial, and fungal communities in our in-silico samples and to understand the elements driving this precision.

### In-silico mock community database construction

**Fig. 1:**
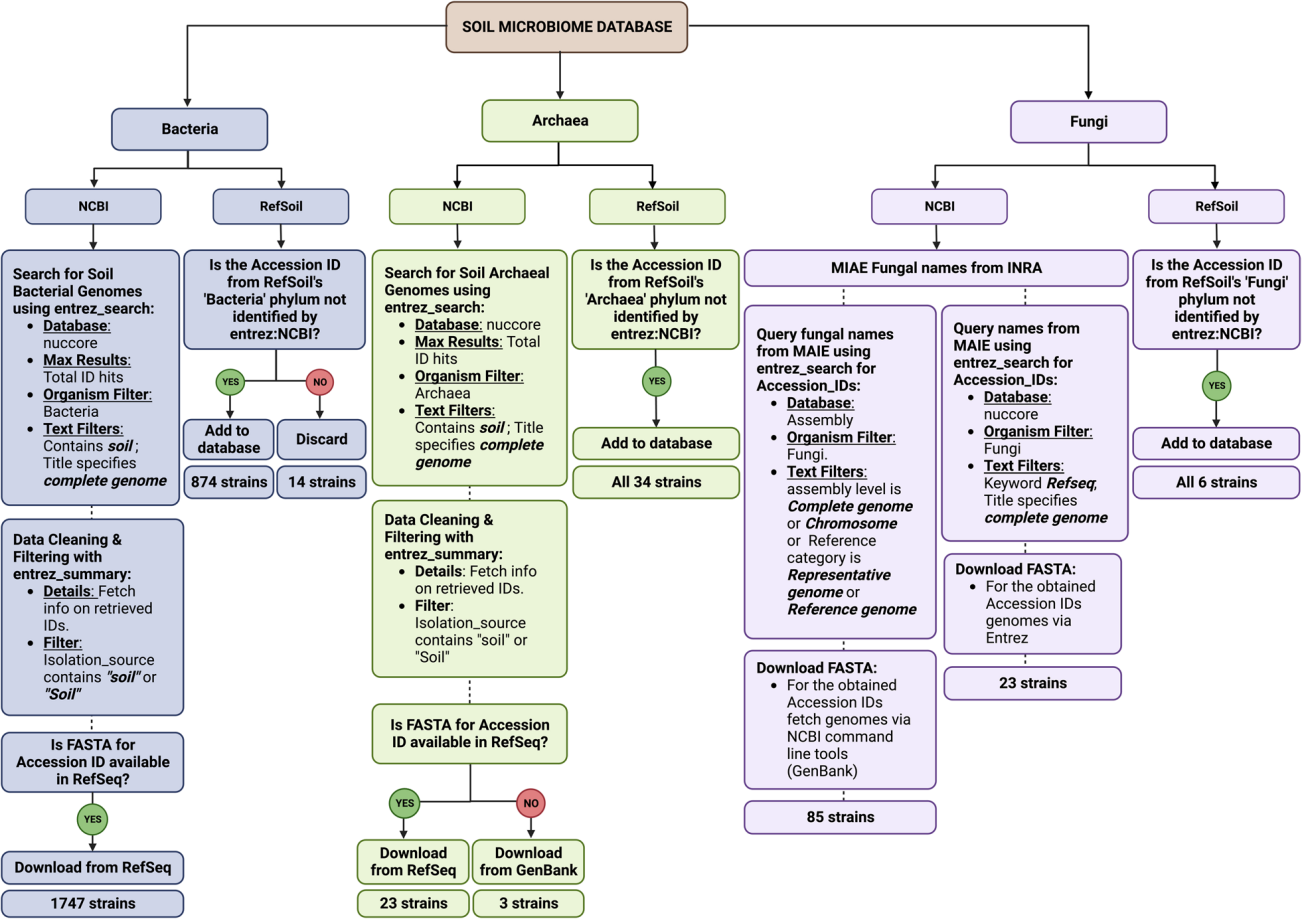
Flowchart detailing the genome selection process for the establishment of a soil microbiome database/mock community. This schematic illustrates the steps and criteria employed to curate genomes of bacteria, archaea, and fungi from various databases

To create the soil-specific database encompassing bacterial, archaeal, and fungal genomes, we combined metadata records from the National Center for Biotechnology Information (NCBI) and the previously published RefSoil database [2]. From the NCBI, we identified bacterial and archaeal genomes that were labelled "complete genome" and specified as isolated from soil. These genomes were retrieved from the nucleotide database as of 14-02-23 using the R package rentrez (R version 4.3.1). While a significant proportion of these genomes were sourced from Refseq, sequences lacking a Refseq representative were supplemented with data from GenBank using the same parameters (Fig. 1). To incorporate soil-derived fungal genomes, we utilized metadata from the MAIE database (INRAE), detailing fungal species isolated from soil, which belonged to phylum *Basidiomycota*, *Ascomycota*, and *Mucoromycota*. While acknowledging that soil's fungal community is more diverse, our inclusion was limited by the available metadata on soil-derived fungal species, compared to bacteria and archaea. FASTA files for these fungi were extracted using NCBI command line tools. Extracted genomes were integrated with the existing RefSoil database, which houses culturable soil-specific organisms spanning bacteria, archaea, and fungi. Finally, we conducted a deduplication process to ensure the retention of unique strains only. The final soil-specific genome database, thus, encompassed a total of 2795 unique strains with 2621 bacterial strains, 60 archaeal strains, and 114 fungal strains. The resulting manually curated database: ‘SoilGenomeDB’ represents both culturable and unculturable soil-specific microbes available in NCBI (Fig. 1).

### In-silico library preparation and sequencing simulations

Ten in-silico NovaSeq sequencing libraries were prepared, with each NovaSeq simulation containing 20 samples, a selection determined to provide a statistically sufficient number of data points across the runs. The number of taxa per sample and the number of reads per sequencing were set using a custom script [26]. Each sample was composed of 1000–25000 genomes, randomly subsampled from the in-silico mock community genome database. This range was chosen to best replicate the inherent complexity of soil environments by incorporating a diverse array of genomes in each sample. Following this, the assignment of reads per sample was executed using the R package ‘EnvStats’ (version 2.7.0). The distribution of reads was set to a truncated log-normal pattern, ranging from a minimum of 5 million to a maximum of 50 million reads per sample, with an average of 16 million reads per sample per run. Once the FASTA files were obtained for each sample, barcodes were introduced using ‘seqkit’ (version 1.4) mutate. Following barcode integration, simulations were then executed using ‘InSilicoSeq’ within the Python3.7 environment [27]. For each sequence in the input FASTQ file, a zero-inflated log-normal distribution was employed, specifically for a paired-end 150-bp NovaSeq run.

### Data quality control

**Fig. 2:**
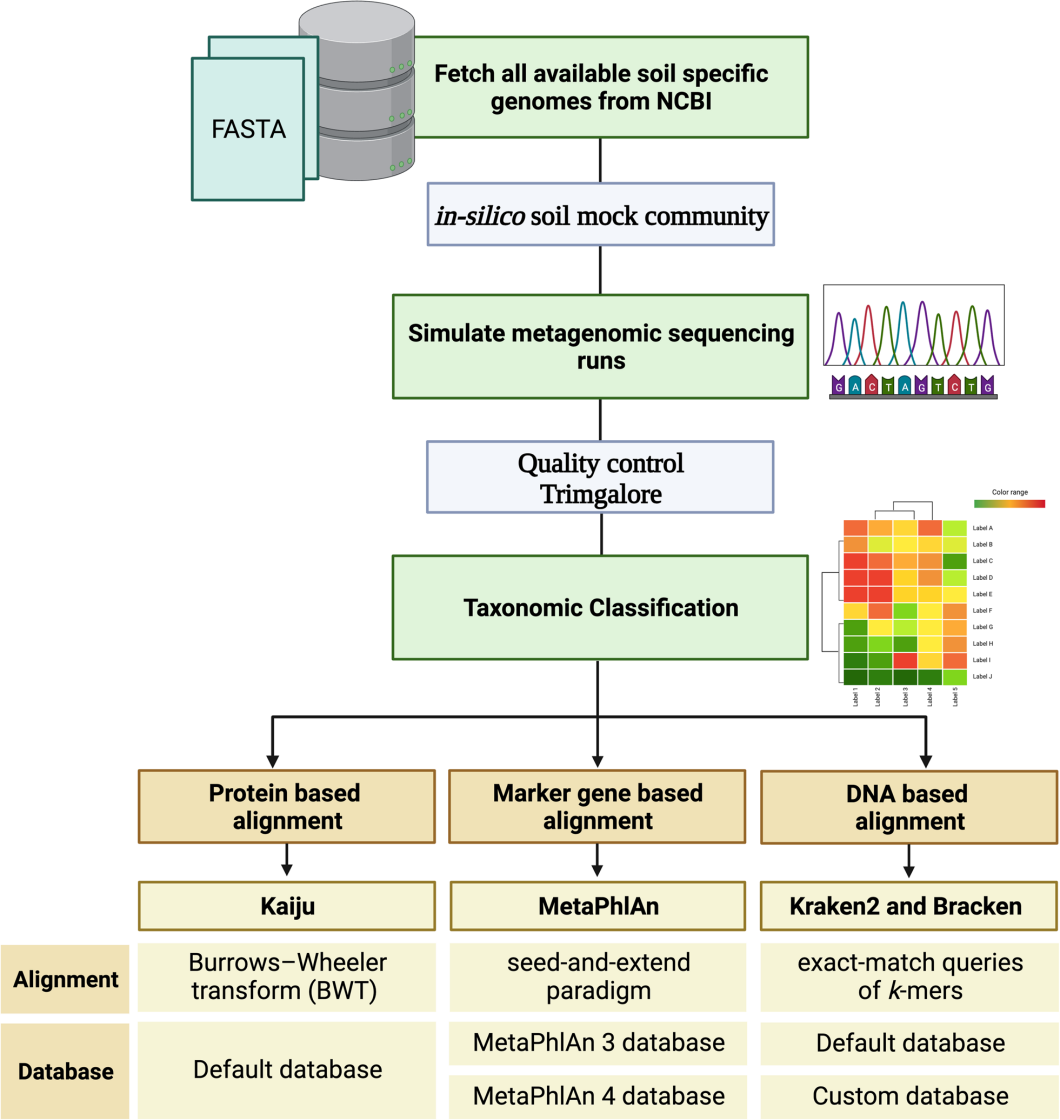
Schematic representation of pipeline used to generate in-silico Illumina data and the taxonomic classifiers compared

Using trimgalore (v0.6.1) barcodes were trimmed from the FASTQ files with Phred score cutoff of 33. A visual representation detailing this procedure can be found in Fig. 2. These trimmed reads were used as the input for the classification of short reads.

Trimmed simulated Illumina reads were assembled into contigs with Spades (v 3.15.3) using flags --meta, -m 500, -t 24, --phred-offset 33, -k 21,33,55,77 [4]. These reads were used as the input for the classification of assembled reads.

### Classifier-Specific Database construction

#### Kaiju

Given the composition of our sample, which was composed of archaea, bacteria, and fungi, we elected to utilize the ‘nr_euk’ database in Kaiju. The ‘nr_euk’ database entails a subset of the NCBI BLAST nr database containing all proteins belonging to archaea, bacteria, viruses, fungi and microbial Eukaryotes (as of 05/03/2023) [28].

#### Kraken2 (default)

The ‘plus-pf’ database was used as the default database for Kraken2. This database consists of complete genomes in RefSeq for bacterial, archaeal and viral genomes, human genome and collection of known vectors (UniVec_Core) plus RefSeq protozoa and fungi (as of 21/07/2022) [29]. A corresponding Bracken database was established, and the bracken abundance profiles were used for analysis [30].

#### Kraken2 (custom)

To create a custom Kraken2 database, all genome_reps from the GTDB repository's latest release (GTDB; latest, 2023-04-27 23:33) were downloaded [31]. The database includes only bacterial and archaeal genomes, hence fungal genomes were downloaded separately. For *Fungal* genomes; all genomes available for phylum *Basidiomycota*, *Mucoromycota*, and *Ascomycota* (as of 6-12-2022) were downloaded from NCBI using NCBI command line tools. A custom Kraken2 and a corresponding Bracken database was then created assigning the downloaded genomes using NCBI taxonomy file. This setup utilized a k-mer length of 39. The Bracken-generated abundance profiles were employed for all subsequent analysis in the current study.

#### MetaPhlAn

Both MetaPhlAn 3 (version 30) and MetaPhlAn 4 (version 4.0) analyses were conducted utilizing the Humann3 module. The specific databases engaged for MetaPhlAn 3 and 4 were mpa_v30_CHOCOPhlAn_201901 and mpa_vJan21_CHOCOPhlAnSGB_202103, respectively [32].

### Benchmarking taxonomic classifiers

The trimmed in-silico FASTQ files underwent taxonomic profiling utilizing the classifiers described above (detailed version control information can be found in Table 1). Each classifier operated using the allocation of 10 CPUs per task. In our study, taxonomic outputs often contained strain-level information, leading to discrepancies across classifier results, especially at the species level. To streamline comparisons, we focused on species-level identifications by retaining only the first two elements (genus and species) of each taxonomic name and removing strain or subsequent details. This approach was particularly relevant for classifiers like Kaiju and Kraken2 (custom), which employ metagenome-assembled genomes (MAGs) having non-standard strain names. We also eliminated special characters to ensure naming consistency, ensuring that species written as 'Pseudomonas sp.' Or 'Pseudomonas sp' were identical for comparison purposes. While this standardization method has its limitations, it was the most transparent way to minimize discrepancies. To further validate our findings, we replicated our tests at the genus level, where naming inconsistencies were less pronounced, confirming the reliability of observed patterns across taxonomic classifications.

**Table 1:** Comparison of taxonomic classifiers: performance metrics, database composition and classification accuracy

Classifier	MetaPhlAn		Kraken2 and Bracken		Kaiju
Version	MetaPhlAn3 v.30	MetaPhlAn4 v.4.0	Kraken2/2.1.1 and Bracken/2.2		Kaiju/1.7.4
Database	CHOCOPhlAn 201901	CHOCOPhlAnSGB 202103	plus-pf	custom database	nr-euk
Organisms included in the database	Bacteria, Archaea, Eukaryota	Bacteria,Archaea, *Microbial Eukaryotes, *Virus	Bacteria, Archaea, Eukaryota, plasmid, human, Univec_core, Protozoa	Bacteria, Archaea, Eukaryota	Bacteria, Archaea, Eukaryota, Virus, Microbial Eukaryotes
Database size	2.4 GB	23 GB	61 GB	1.2 TB	144 GB
Processing time per sample (h:m:s)	3:02:38	4:24:14	0:43:29	2:20:08	11:23:26
Species F1 score (optimal threshold)	0.26 ± 0.02	0.41 ± 0.02	0.74 ± 0.01	0.68 ± 0.0	0.48 ± 0.01
Species F1 score (no threshold)	0.26 ± 0.02	0.42 ± 0.02	0.5 ± 0.01	0.63 ± 0.01	0.11 ± 0.01
Unclassified	94.5%	90.6%	79.3%	0.46%	37.43%
Classified	5.5%	9.4%	20.7%	99.54%	62.57%

*MetaPhlAn4's database primarily encompasses bacterial and archaeal sequences, with limited coverage of viral and eukaryotic microbial sequences [32]

The taxonomic profiles generated by each classifier at various relative abundance thresholds were evaluated based on several metrics: F1, precision, sensitivity, and Bray–Curtis community composition. Additionally, the Euclidean distance metric was employed, and the Wilcoxon test, Kruskal Wallis test and Dunn tests were utilized to assess the significant impact of various factors on the results.

Using the F1 scores and Euclidean distances as a preliminary evaluation, the in-silico assembled contigs were then compared. Contigs were processed with the top three classifiers from the initial evaluation: Kraken2 (default), Kaiju, and the Kraken2 (custom). We assessed these classifiers by comparing their F1 score, Precision, and Sensitivity. This comparison was conducted on two levels: between the taxonomic classification of the contigs and the classification of quality-trimmed reads, as well as against quality-trimmed reads at the identified relative abundance threshold.

### Statistical analysis

Statistical analysis was performed in R 4.3.1. The vegan package (version 2.6-4) was used for Bray–Curtis-based multidimensional scaling (MDS) analysis and calculating Euclidean distances. The analysis of variance using distance matrices (adonis2) function in vegan with 999 permutations was used for PERMANOVA (permutational analysis of variance) analysis. The Kruskal–Wallis test and Wilcox tests were performed in base R to identify significant differences, and the resultant p values were adjusted using the Bonferroni method. All the R packages and versions utilized in the paper are described in Additional file 2: Table S1.

### Accuracy metrics

To ascertain the accuracy with which the taxonomic classifier captured the microbial profile of the in-silico shotgun community at the species and genus level, the critical metrics including Precision, Sensitivity, and the F1 score (as indicated below) were utilized to provide insights into the comparative performance of the classifiers.

Precision (Positive Predictive Value) quantifies how many of the predicted positive classifications were correct.

$$Precision = \frac{True\,Positives}{{True\,Positives + False\,Positives}}$$

Sensitivity (Recall) measures the proportion of actual positives that are correctly identified.

$$Sensitivity = \frac{True\,Positives}{{True\,Positives + False\,Negatives}}$$

F1 score is the harmonic mean of precision and sensitivity, which provides a single metric that encapsulates the balance between precision and sensitivity. It is particularly useful when the data has imbalances.

$$F1\,score = 2 \times\frac{{\left( {Precision \times Sensitivity} \right)}}{Precision + Sensitivity}$$

### Real environmental metagenome dataset

We aimed to assess whether the implementation of the best-performing classifier would alter our understanding of previously published data. To do this, we chose a soil metagenome dataset from a 2021 publication available in the NCBI Sequence Read Archive (SRA) by Mantri et al. [33]. The dataset met the following requirements: (i) originated from a soil matrix, (ii) sequenced using Illumina NovaSeq, (iii) raw sequencing files were publicly accessible on the SRA with detailed metadata, and (iv) the original study utilized at least one of the classifiers under investigation. This dataset, closely reflecting our simulated conditions, encompasses seven soil metagenomes.

We conducted a reanalysis of the environmental soil metagenomic dataset, which was previously examined using maxikraken (available from https://lomanlab.github.io/mockcommunity/mc_databases.html), by applying Kraken2 (custom) to the published shotgun data for a more detailed investigation. Our analysis was then compared to the original published results at the phylum level. It is worth noting that we recreated the published figure using their taxonomic classification results, but with updated phylum names. A comparison between the original figure, which uses the old phylum names, and our rendition can be found in the Additional file 1: Fig. S6.

## Results

We compared the performance of several taxonomic classifiers: Kaiju, Kraken2 with Bracken, MetaPhlAn 3, MetaPhlAn 4, and a custom version of Kraken2 with Bracken using a database derived from GTDB-TK genomes on our in-silico samples. The analysis aimed to determine the bacterial, archaeal, and fungal composition of the in-silico shotgun metagenomic samples.

The DNA-to-Marker technique, applied by MetaPhlAn3 measures taxon genome counts relative to the total detected genomes. In contrast, Kraken2 operates on a DNA-to-DNA basis, and Kaiju utilizes a DNA-to-Protein approach. Both aim to estimate the proportion of assigned sequences, thus emphasizing sequence abundance over taxonomic abundance. Each classifier, while covering bacteria and archaea universally, relies on databases with unique taxonomic inclusions. Kaiju's “nr-euk” database integrates viruses, microbial eukaryotes, and eukaryota; MetaPhlAn3 includes Eukaryota; and Kraken2 “plus-pf'' adds viruses, plasmid, Univec_core (for vector contamination screening), and protozoa. Our Kraken2 (custom) database further incorporates phyla *Basidiomycota*, *Ascomycota*, and *Mucoromycota* to reflect our in-silico community and to partially capture the fungal diversity in soil. For a concise overview of each classifier's characteristics and performance metrics, see Table 1. In this section, we delve into the implications of relative abundance threshold, classifier selection, and contig assembly in taxonomic classification.

### Influence of relative abundance threshold

Initially, we evaluated whether the introduction of a minimum relative abundance threshold impacted the overall performance of each classifier at species and genus level classification, using classic model performance metrics such as considering the F1 score, sensitivity, and precision.

**Fig. 3:**
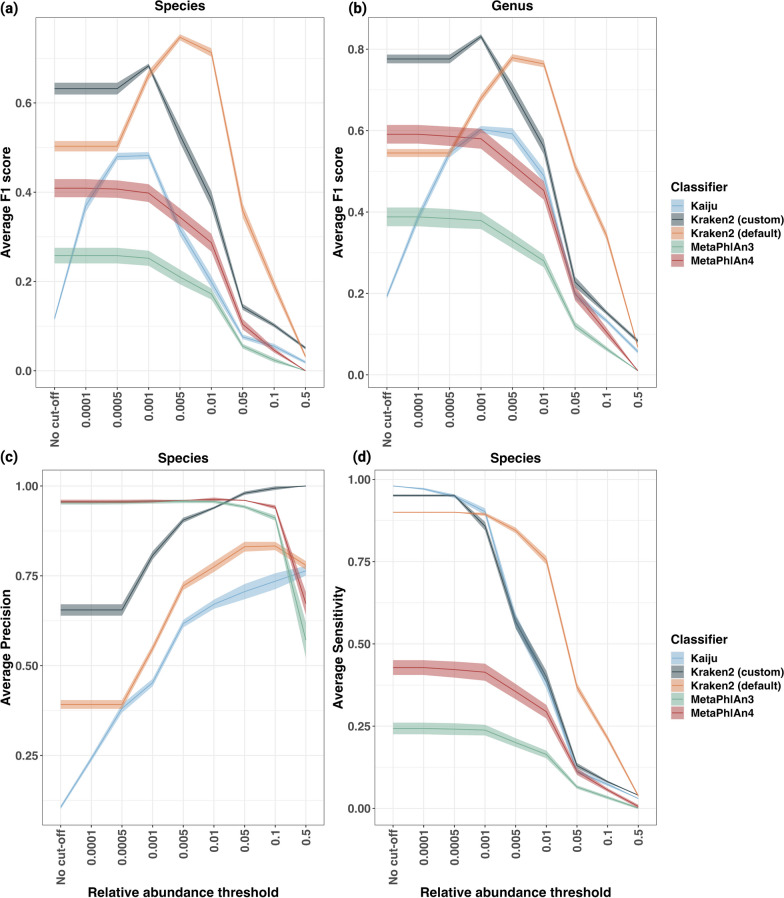
Average F1 scores at species and genus levels (**a** and **b**, top row) alongside precision and sensitivity metrics at species level (**c** and **d**; bottom row) for various classifiers on the in-silico dataset at different relative abundance thresholds. Each plot displays the median with ribbon plots indicating the range of standard deviation

In assessing classifier performance, Kraken2 (both default and custom) and MetaPhlAn's F1 scores remained stable at a low relative abundance threshold for both species and genus levels (Fig. 3). Most classifiers demonstrated optimal performance at a relative abundance threshold of 0.001% with Kraken2 (custom) registering an F1 score of 0.68 + / − 0 and MetaPhlAn3 at the lower end with 0.26 + / − 0.02. Kraken2 (default), however, reached its highest performance at 0.005% with an F1 score of 0.74 + / − 0 0.01. Beyond these optimal points, F1 scores began to diminish. Overall this indicates that an optimal relative abundance threshold varies by classifier and database combination. This is important as certain classifiers and databases excel at identifying species that are present in low quantities, which can be crucial in soil metagenomics, where low-abundance organisms can play fundamental roles in the dynamics of microbial communities and ecosystems.

To investigate the factors affecting F1 scores, we examined precision and sensitivity by species. Among classifiers, Kraken2 (custom), Kraken2 (default), and Kaiju often provided a high number of false positives, resulting in reduced precision. However, their robust sensitivity, characterized by minimal false negatives (see Fig. 3c, d; Additional file 1: Fig. S1a, b), compensates for this shortfall in precision. On the other hand, MetaPhlAn 3 and 4 performed with acceptable precision but poor sensitivity, placing them at a relative disadvantage when compared to the other classifiers (Fig. 3).

We identified significant differences in the performance metrics F1 score across different classifiers and different thresholds (p-value < 0.001). These differences became particularly noticeable when we grouped the thresholds and compared the performance of classifiers against each other. Dunn's post-hoc test indicated that MetaPhlAn's performance was significantly different to other classifiers. A higher z-value means that the two groups are further apart in terms of standard deviation. In our results, Kraken2 (default) vs. MetaPhlAn3 had a z-value of 9.05 (p-value < 0.001), Kraken2 (custom) vs. MetaPhlAn3 with a z-value of 7.63 (p-value < 0.001), Kraken2 (default) vs. MetaPhlAn4 with a z-value of 5.6 (p-value < 0.001), and Kraken2 (custom) vs. MetaPhlAn4 z = 4.2 (p-value < 0.001). Comparing MetaPhlAn3 and MetaPhlAn4, a difference with z = 3.4 (p-value < 0.01) was observed. In contrast, no significant differences were noted between Kaiju and both MetaPhlAn3 and 4. These findings highlight MetaPhlAn's markedly different performance from the other classifiers evaluated (Table 1).

Regarding relative abundance thresholds, a post hoc Dunn test—applied across all classifiers and samples to isolate the impact of threshold values—indicated that the values at extreme thresholds tested, notably 0, 0.05%, 0.1%, and 0.5%, exhibited significant variations in F1 score when compared to other threshold settings (Additional file 2: Table S2). Balancing precision and sensitivity necessitates the selection of optimal classifiers and thresholds for soil shotgun metagenomic data. Our analysis identifies the optimal relative abundance threshold of 0.001% for Kraken2 (custom), Kaiju, and MetaPhlAn, and 0.005% for Kraken2 (default) at the species level (Fig. 3).

### Role of taxonomic classifiers in estimating species-level relative abundance

**Fig. 4:**
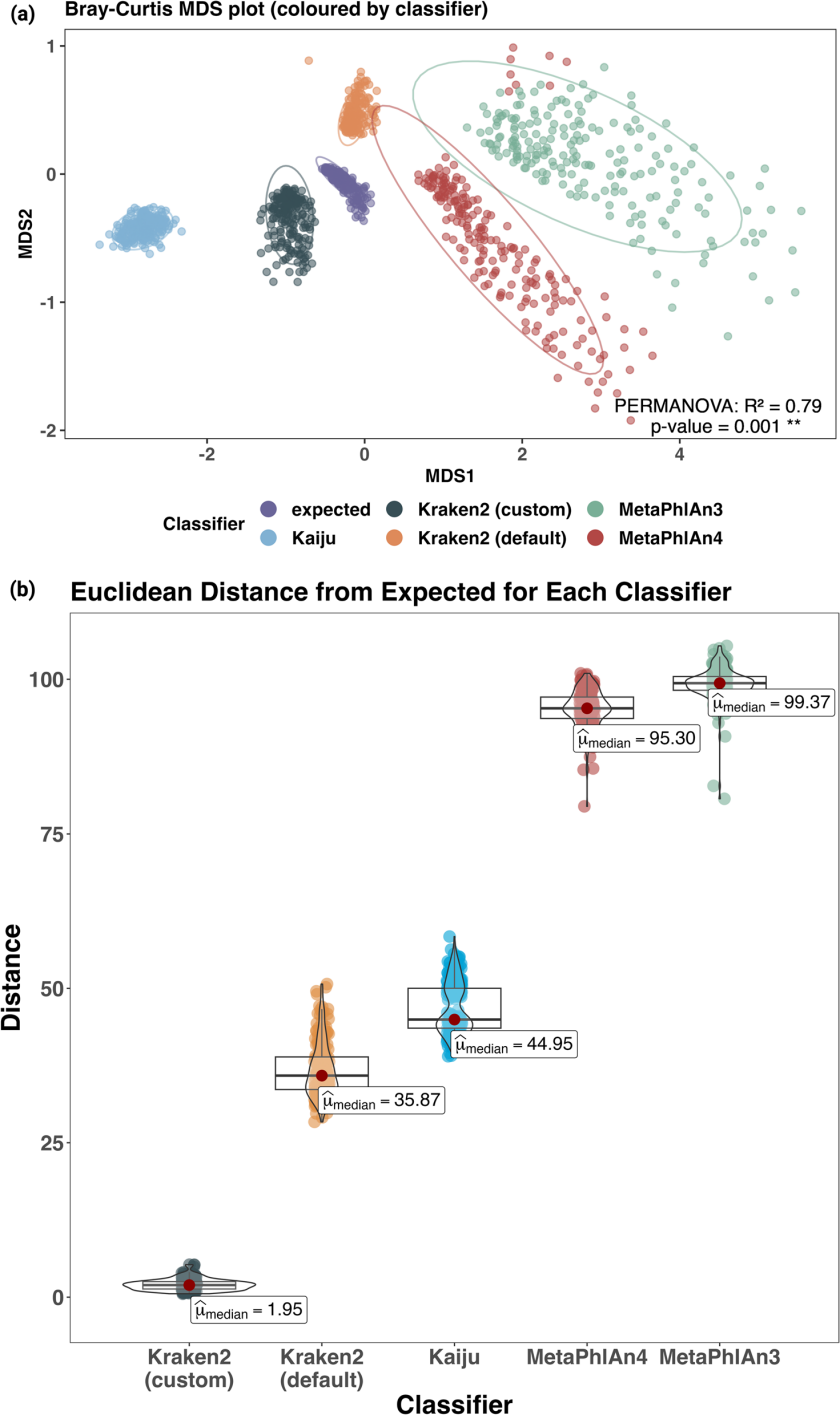
Compositional analysis of in-silico data across classifiers. **a** Dissimilarity plot illustrating species-level community profiling deviations from expected among different classifiers. **b** Boxplot of Euclidean distances comparing observed to expected species level relative abundance for different classifiers. Both visual representations indicate that Kraken2 (custom) offers the closest approximation to the epected species community profile

By using the optimal relative abundance threshold, we examined the community structure and tested whether species' relative abundances were deviating from expected values. To do this, we employed Multidimensional Scaling (MDS) to identify which classifiers could accurately discern the in-silico community structure and report relative abundance values that align with expected values. Applied to the compositional data, MDS confirmed marked variations between different classifiers (PERMANOVA: p-value = 0.001, R^2^ = 0.79) (Fig. 4a). The classifier accounted for approximately 80% of the observed variance in microbial community composition.

In assessing classifier accuracy at the species level using the Euclidean distance between observed and expected outputs, Kraken2 (custom) consistently demonstrated superior performance in comparison to other classifiers. The Kruskal–Wallis tests, conducted to compare distance values among different classifiers across the runs, consistently pointed to significant differences. Specifically, for each run, the effect size (H-value) was large, ranging from approximately 0.902 to 0.950. The p-values for all runs varied between 0.05 to 0.001, emphasizing the robustness of these results (Additional file 2: Table S3a). A post-hoc Dunn test with Bonferroni adjustment demonstrated that Kraken2 (custom) differed significantly from Kaiju, MetaPhlAn4, and MetaPhlAn3 across all 10 runs (p-value range < 0.001–0.01], H-value range: [3.92–8.6]; Additional file 2: Table S3b). In these runs, Kaiju also significantly varied from MetaPhlAn3 (p-value range < 0.001, H-value range: [3.97–4.47]) and exhibited differences from MetaPhlAn4 in one run (p-value: < 0.01, H-value [2.93]). The distinctiveness of Kraken2 (default) from both MetaPhlAn3 and MetaPhlAn4 was evident throughout the ten runs. Figure 4b provides a visual representation of these average distances for each classifier in the form of a box plot.

Understanding the unique methodologies employed by each classifier is crucial. While MetaPhlAn3 utilizes a DNA-to-Marker technique, emphasizing taxon genome counts, Kraken2 and Kaiju underscore sequence abundance over taxonomic distinction via their DNA-to-DNA and DNA-to-Protein strategies, respectively. Notably, when no cut-off was applied, MetaPhlAn3's results substantially deviated from expected values. Nevertheless, the Euclidean distances for MetaPhlAn3 remained consistent, regardless of the relative abundance threshold of 0.001. Such uniformity of MetaPhlAn suggests the relative abundance threshold limited influence on the taxonomic abundance precision, which is supported by comparable F1 scores at both levels (Table 1).

### Comparative efficiency of classifiers in metagenomic short-read utilization

In our analysis comparing the efficiency of various taxonomic classifiers in read classification, we found that on average, across ten separate runs, the classification rate was 99.54% with Kraken2 (custom), 63% with Kaiju, 21% with Kraken2 (default) and 5–10% with MetaPhlAn (Table 1) in diverse simulated sequencing libraries. This underscores the superior efficiency of Kraken2 (custom) in maximizing sequencing output value. While one might assume a high percentage of unclassified reads indicates a vast array of unidentified species, it's essential to recognize that this can often be a consequence of the selected classifier and database. Therefore, these figures might not truly reflect the sample's diversity, but rather the limitations or specificity of the analytical tools used. Although Kraken2 (custom) provides the closest values (very low Euclidean distance) to the expected relative abundance values at the species level, it is not statistically significant from Kraken2 (default). Despite their similar performance in identifying relative abundance values, Kraken2 (custom)vastly outperforms Kraken2 (default) by classifying 99% of reads compared to Kraken2 (default) 21%. This underscores a distinct discrepancy in their ability to analyse sequencing data, clearly attributed to the underlying databases each employs, given the constant classifier and algorithm.

### Assembled vs. unassembled reads: evaluating the optimal approach

Contigs, with their longer sequences and assembly from multiple reads, promise enhanced specificity in taxonomic classification. Their historical relevance and analytical efficiency further positioned them as potential candidates for taxonomy assignments. To analyse this we selected five random runs from the sequenced data to assemble with Spades, followed by classification using the three top-performing classifiers; this facilitated a detailed comparison between the default unfiltered taxonomic profiles, those adjusted to their optimal relative abundance thresholds, and the taxonomic profile of the assembled contigs. Using the Kruskal–Wallis test, we compared the F1 scores of taxonomic classifications on assembled contigs versus trimmed reads (at optimal relative abundance threshold for species-level classification). For both versions of the Kraken2 (default and custom) classifier implementation, the observed F1 score was lower for assembled contigs than trimmed reads with and without a relative abundance threshold filter. Notwithstanding this, the relative abundance threshold in conjunction with Kaiju favoured contigs. From the perspective of classifier performance, we observed significant variations in F1 scores between the two read types: Kaiju (p-value < 0.01), Kraken2 (default) (p-value < 0.01), and Kraken2 (custom) (p-value < 0.01). However, upon applying optimal relative abundance threshold filters, trimmed reads consistently outperformed contigs across all classifiers in terms of F1 scores and precision. Notably, sensitivity declined at these optimal relative abundance thresholds, suggesting an increased likelihood of false negatives (Additional file 1: Fig. S2). In summary, the use of assembly-based methods to assess taxonomic diversity resulted in a reduction in classification accuracy.

### Evaluating taxonomic misclassifications across different classifiers

While our efforts have been directed towards ascertaining the superiority of specific classifiers under given conditions, it is equally crucial to investigate what remains undiscovered or erroneously identified in the course of our investigations.

The chi-square statistical analysis substantiated a significant divergence in the family-level taxonomic classifications generated by the four evaluated classifiers, reflecting notably distinct patterns in the identification of true positives, true negatives, false positives, and false negatives across the families picked by each classifier (χ^2^ statistic = 135,543–4395.4, p-value < 0.001) [detailed in Additional file 2: Table S4 and illustrated in Additional file 1: Fig. S3]. The divergence suggests that the observed taxonomies at the family level are profoundly influenced by the choice of classifier, rather than being an accurate representation of the actual sample profile. This finding emphasizes the need for researchers to treat the interpretation of taxonomic data with caution, as the choice of classifier can have a significant impact on the biological interpretations and conclusions that are drawn.

**Fig. 5:**
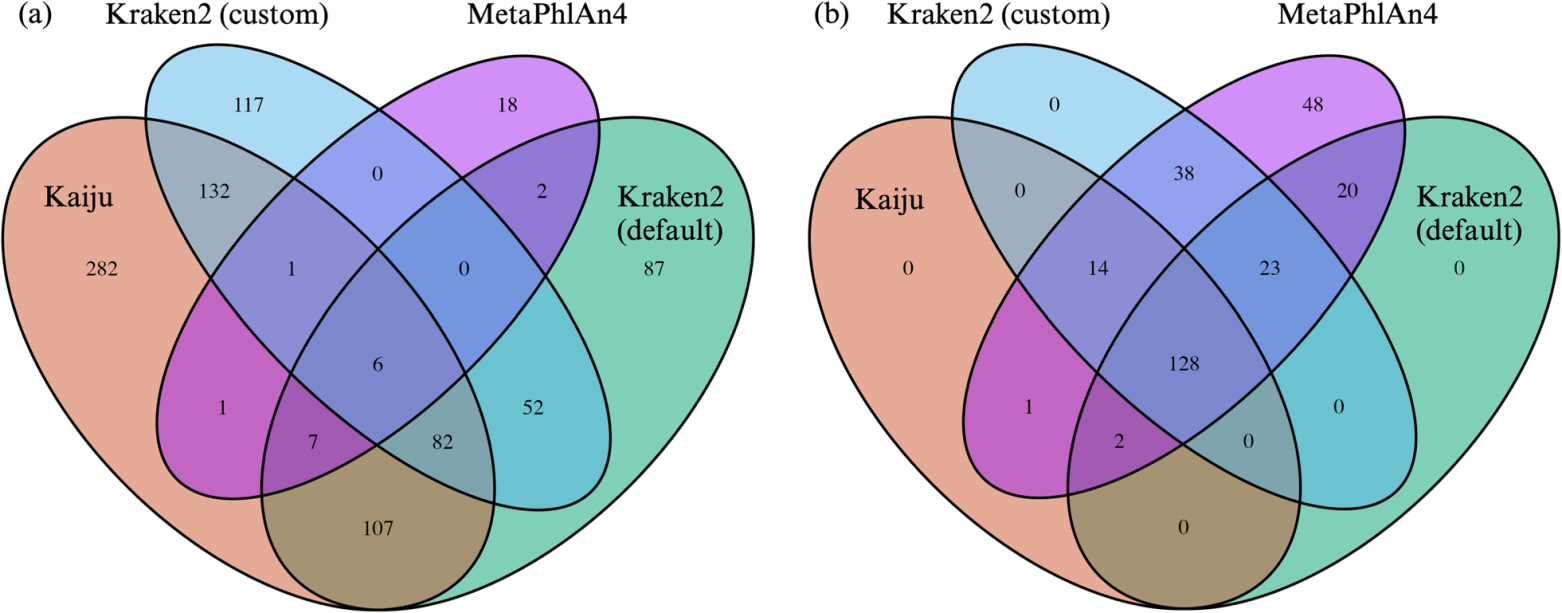
Venn diagram illustrating differential false positives (**a**) and false negatives (**b**) by classifiers at family level. The Venn diagrams show an inverse relation between FP’s and FN’s. The Kraken2 (custom), Kraken2 (default), and Kaiju lead in FP’s but have minimal FN’s, often aligning with MetaPhlAn4. Meanwhile, MetaPhlAn4 exhibits fewer FP’s but more unique FN’s

To further examine the distribution of false positives (FP’s) and false negatives (FN’s) at higher taxonomic categories like family level, we employed Venn diagrams and heatmaps. The Venn diagrams depict an interesting inverse relationship between FPs and FNs. Notably, while Kraken2 (custom), Kraken2 (default), and Kaiju are major contributors to FPs, their influence on FNs is considerably smaller, often overlapping with MetaPhlAn4. Conversely, MetaPhlAn demonstrates fewer unique FPs but a larger number of unique FNs (Figs. 4b and 5a).

Heatmaps emphasize the divergence amongst classifiers in terms of false positive detection. Kaiju adeptly identified Fungi without registering any FNs, yet provided a significant number of FPs specifically for fungal families. Conversely, Kraken2 (default) exhibited the opposite trend with fewer fungal FPs but a notable number of FNs. An increase in FPs can be observed when the underlying database has sequences outside the mock community's makeup, leading to misclassifications in kingdoms like *Viridiplantae*, *Chromista*, *Metazoa*, and *Viruses.* Kaiju's misclassifications and Kraken2 (default) consistent errors in the *Chromista* and *Hominidae* families further illustrate this. To improve accuracy in sequence data analysis, it might be beneficial to use databases mainly consisting of sequences from the targeted microbes, specific to the research environment (for instance, excluding terrestrial mammal sequences in marine microbiome studies). This approach could enhance classification precision, although it may narrow the scope of the investigation. Kraken2 (custom), however, strikes a balance with the fewest FPs but misses certain families like *Bdellovibrionaceae* and *Rhodobacteraceae* (see Additional file 1: Fig. S2; Fig. 3).

In terms of true positives, MetaPhlAn4 predominantly detected common family hits, while Kraken2 (default), Kraken2 (custom), and Kaiju had additional shared detections. The varied counts among classifiers, with Kraken2 (custom) and Kaiju spotting unique families, highlight the differential efficiency of these tools in true positive identifications (Additional file 1: Fig. S1(d)).

### Refining biological insights with optimized taxonomic classification

In our analysis of the NovaSeq soil dataset, we utilized the Kraken2 (custom) classifier, informed by our earlier in-silico evaluations. The real metagenome dataset used for this study was obtained from a previous study by Mantri et al. 2021 [1] encompassing seven soil metagenomes from samples collected from soil horizons of multiple forest sites in Germany. Our comparative analysis with the original study, which employed maxikraken, revealed a notable difference in both the identified phyla and the classification of reads. Our analysis resulted in an average of 58% classified reads, a marked improvement from the 47.04% reported in the original study. This variation in read classification percentages underscores the inherent challenge of microbial identification in complex soil matrices. Nevertheless, both methods identified prominent soil phyla such as *Planctomycetota*, *Actinomycetota*, *Chloroflexota*, *Pseudomonadota*, and others.

**Fig. 6:**
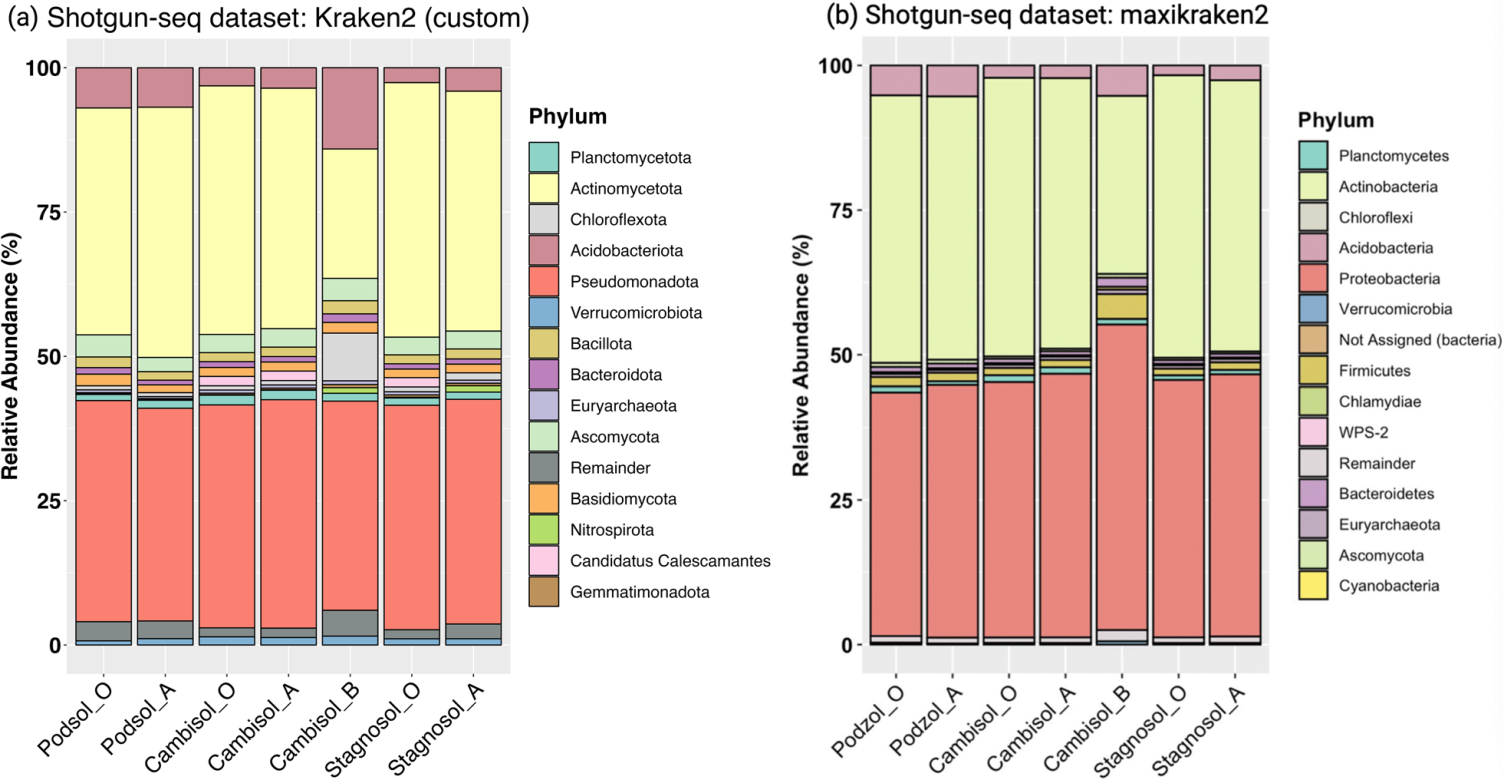
Comparative microbial composition across three sampling sites and soil horizons at the phylum Level. **a** Bar plot representing the taxonomic profile of the shotgun dataset using Kraken2 (custom). **b** Bar plot illustrating the taxonomic profile of the shotgun dataset as derived from the original study. Both plots showcase the top 14 phyla with distinct colours, while the remaining phyla are collectively grouped as “Remainder”. Consistent colour schemes are used across both plots for the same phyla. The x-axis delineates the three sampling sites (Podsol, Cambisol, and Stagnosol) and is further segmented into the three soil horizons (O, A, and B) for each site

Two deviations were prominent. Cambisol_B had a higher number of reads assigned to *Chloroflexota* presence with Kraken2 (custom) compared to maxikraken results. Despite similar levels of *Actinomycetota* and *Pseudomonadota*, we detected increased proportions of *Acidobacteriota* and *Ascomycota* across all samples. Our reanalysis, utilizing the Kraken2 (custom) also enabled the identification of several phyla—*Gemmatimonadetes*, *Candidatus Calescamantes* and *Nitrospirota*—which notably emerged among the top phyla in our study, but were not identified as prominent phyla in the original study (Fig. 6).

The beta diversity of microbial communities across seven soil samples reaffirmed our *in-silico* observations. On examining species-level distinctions, the NMDS plot revealed that the Kaiju and Kraken2 (custom) tools tended to cluster together, potentially due to the shared utilization of Metagenome Assembled Genomes (MAGs) in their respective databases, as depicted in (Additional file 1: Fig. S7(a)). Additionally, PERMANOVA (adonis2) analysis indicated that both the sample site (R2 = 12.3%, p < 0.01) and the taxonomic tool used (R2 = 85.09%, p < 0.001) influenced diversity at the species level. Additional PERMANOVA (adonis2) analysis was conducted to determine whether this was also observed at the genus level. Consistent with the species-level findings, the results affirmed the significant impact of both the sample site and the taxonomic tool on genus-level diversity, explaining 18.46% (p = 0.001) and 77.5% (p = 0.01) of the variance, respectively, as illustrated in (Additional file 1: Fig. S7(b)). Clearly, classifiers have a stronger influence on microbial profiles than the inherent soil composition, once again emphasizing the importance of benchmarking.

## Discussion

In this study, we employed the latest versions of three metagenomic taxonomy classification tools: MetaPhlAn, Kaiju, and Kraken2. Our findings illustrate that variations in classification tools, parameters, and databases can substantially affect both the percentage of classified reads and the diversity of identified species, as detailed in Table 1. Here, we aimed to enhance the accuracy of species-level classification, reflecting the true complexity of soil microbiomes. We analysed different relative abundance thresholds, classifiers, and input data types (contigs or trimmed reads) using various simulated soil datasets. Our findings indicate that the Kraken2 tool was most effective when combined with Bracken at a relative abundance threshold of 0.001% (Figs. 3 and 4). This optimum performance was further enhanced by selecting a database that encompasses the pertinent kingdoms under investigation and utilizing trimmed reads for classification (Additional file 1: Fig. S2).

In line with our in-silico evaluations, the use of Kraken2 (custom) in analysing real-world NovaSeq soil dataset [33] led to distinct findings compared to the original study using maxikraken. Specifically, our analysis yielded an average of 58% classified reads, an improvement over the 47.04% in the original study that utilized maxikraken. Additionally, we identified key phyla, including *Gemmatimonadetes*, *Nitrospirota*, *Basidiomycota and Candidatus Calascamantes*, which did not appear in the original study's list of dominant phyla (Fig. 6). The elevated presence of the *Gemmatimonadetes* phylum in our data aligns with findings from other research where it was recognized as a significant component of the forest soil microbiome [34, 35]. Further, the Nitrospirota phylum, which is prominently represented in our dataset, has been highlighted in recent research for its role in soil nitrification through complete ammonia-oxidizers. Importantly, beta diversity analysis affirmed the influence of the classifier choice on microbial profiles, even exceeding the effects of soil composition (Additional file 1: Fig. S7(a)).

In line with previous research, MetaPhlAn underperformed when applied to soil microbiome data, thereby suggesting it might be unsuitable for analysing soil metagenome data [32]. Echoing the findings of Ye et al. [36] and Govender et al. [37], our research found that the DNA-to-DNA classifier, notably the combination of Kraken2 and Bracken, delivers superior precision, recall, and abundance estimates compared to the DNA-to-protein-based classifier (Kaiju) and the marker-gene classifier (MetaPhlAn). In the context of our study, the improvement in the F1 score as well as the Euclidean distance we observed by utilizing the Kraken2 classifier may have been potentially influenced by Bracken's capability to redistribute reads from intermediate taxonomic levels, as suggested by previous studies using Illumina data [30, 36].

In addition, our findings differ from prior studies, particularly regarding minimum relative abundance thresholds and the use of assembled contigs for taxonomic analysis. The current study observed a decline in F1 score at relative abundance thresholds of 0.5% and 0.1%, in contrast to the recommendations made by Ye et al. 2019. Although the filtration of low-abundance taxa has been advocated to mitigate false positives in shotgun metagenomic data [14, 19], our findings indicate that higher thresholds could potentially neglect critical low-abundance taxa integral to soil microbiomes. The ecological importance of rare taxa has recently been highlighted [37–39] and keeping a high relative abundance threshold might limit the sensitivity of the analysis. Similarly, while previous studies recommend the use of assembled contigs [20], our study found that utilizing trimmed reads yielded more accurate taxonomic analyses. Discrepancy may result from difficulty in assembling contigs when high strain diversity and low abundance species are present in a sample, leading to low coverage of strains [40]. In many instances, assembly algorithms discard these rare genomic fragments, thereby failing to accurately capture these elusive taxa. Thus, bioinformatic guidelines must be applied carefully, taking into account the ecological complexities of different samples such as soil or marine environments, rather than directly adopting strategies based on clinical studies. Given these observations, we suggest employing lower relative abundance thresholds, precisely 0.001 or 0.005, during the analysis of highly complex microbial communities from soil samples to ensure more accurate results.

Interestingly, our study revealed that popular classifiers such as Kraken2 (default) (database: “pluspf”), Kaiju (database: “nr-euk”), and MetaPhlAn3 and 4, left a substantial proportion of reads unclassified—even using an in-silico sequenced data generated from known NCBI genomes. This observation challenges numerous studies where high levels of unclassified reads have been interpreted as indicators of microbiome novelty. The limitations of these classifiers further underscore the importance of in-silico studies, which provide a controlled environment for benchmarking.

A critical challenge in benchmarking studies is the continual evolution of the NCBI taxonomic database. The classifiers Kaiju and Kraken2 (custom) used in the current study incorporates Metagenomic Assembled Genomes (MAGs) in their underlying databases, which currently feature unique, transitioning, species identifiers facilitated through SeqCode [41–43]. There is a necessity to carefully select the database in alignment with the specific sample at hand; particularly, soil matrices which harbour a multitude of uncultured organisms that might necessitate the integration of MAGs in their analysis, to ensure heightened classification accuracy [44]. A future study could explore the impact of adding MAGs to the databases on classification accuracy since emerging evidence suggests that MAGs can contribute significantly to the exploration of highly complex ecosystems such as soils [44–46]. Compounding this challenge is the current reliance of Kraken2 databases on RefSeq [47], which presently does not incorporate MAGs, potentially hindering performance in environments with low-resolution databases like the soil we have investigated here. This indicates a potential need for custom databases to enhance classification accuracy, despite Kraken2's notable effectiveness as a classifier (Fig. 4) [36, 48].

### Limitations of the current study

A significant constraint of the present study is the variation in the underlying databases applied in the different classifiers, which impedes a direct comparison to ascertain the best classification algorithm for the analysis. Creating such expansive databases with specific classifiers presented significant technical hurdles. This limited the application of other powerful tools like DIAMOND [49] and Kaiju [28]. Constructing a large custom database using GTDB-TK genomes illuminated that there was a clear cost–benefit trade-off between the comprehensive scope of the database and the efficiency of the classifier. Although it was easy to set up a custom database with DIAMOND, its lengthy runtime per sample—about a day on average—made it impractical for our study (for a detailed comparison of classifier timings please refer to Table 1. Whereas, developing a custom database for Kaiju demanded considerable computational resources, bioinformatics expertise, and time, constraints that rendered it unfeasible for us to accomplish in this study. Overall, our experience underscored the accessibility and user-friendliness of Kraken2, not only as an optimal choice with a customized database but also proving its efficacy with the default database, which emerged as the second-best performer in our study. This dual success establishes Kraken2 as a highly preferable option for bioinformatics beginners, offering a less technically demanding pathway to obtain reliable and precise results.

A second concern in metagenome analysis is the database and the bias inherent in each classifier. In our study, a notable anomaly was the high incidence of false positives for human contamination reported by Kraken2 (default)—averaging at 14.9% (+ / − 4.83 SD) at the genus level—when using its default "plus-pf" database. While a recent study indicated that parts of the human genome mistakenly categorized as bacteria in the NCBI could cause false identifications [50], our observations suggest that in our case, the increased false positives are primarily due to the inclusion of human genomic reads in the Kraken2 (default) database, an issue not identified with other classifiers as they do not incorporate human genomes in their database. Hence, researchers must exercise caution when analysing the results from Kraken2 to avoid incorrect associations with human genomic signals.

It is important to note that while our study focuses on the differences in taxonomic classification tools using real soil samples, these samples were collected from forested areas in close proximity to each other. Observing the substantial impact of classifier choice in this context, we postulate that classifier choice would manifest similarly across varied soil environments including those in arid, grassland, and arctic regions. We hypothesise a dual-layered clustering where environmental characteristics and the classifier used would significantly influence microbial profiling. We anticipate that samples would cluster both by their environmental context and by the taxonomic tool employed, with the latter possibly being the dominant clustering factor. However, this hypothesis is extending beyond our study's scope, and future research is needed to explore this dual influence and to validate the hypothesis across diverse ecosystems.

## Conclusion

In summary, the accuracy of microbial taxonomic profiling largely depends on the choice of classifier and database; often, "unclassified" results may be due to limitations in these tools rather than true representations of environmental novelty. Kraken2 (custom) deployment in the real data set revealed distinct findings of taxa not picked by maxikraken, and given our benchmarking, stands as a dependable tool for shotgun metagenomics of the soil microbiome. In situations where computational resources are limited, utilizing Kraken2 with an updated database and adjusting parameters to match the study's objectives can be a viable alternative. Our findings demonstrate the pressing requirement for more comprehensive benchmarking studies, which are tailored to accommodate the distinct characteristics of varying environments or sample types.

### Supplementary Information

**Additional file 1. Fig S1:** Venn diagrams depicting classifier disparities at the species level for false positives (a), false negatives (b), and true positives (c). Differential true positives at family level are shown in (d). **Fig. S2:** Comparison of F1 score, sensitivity and precision for different taxonomic classifiers on assembled reads and QC/ trimmed reads. **Fig. S3:** Balloon plot visualization of classification outcomes at the family level using different tools. The size of each balloon corresponds to the count of observations under specific outcome categories: FN (False Negatives), FP (False Positives), TN (True Negatives), and TP (True Positives). The colour gradient within the balloons indicates the magnitude and direction of deviations between observed and expected classifications. Numbers displayed on the right or bottom of the plot represent cumulative counts across respective categories. **Fig. S4:** Heatmap representing consistently misclassified families. This heatmap displays the family level classification that were identified as false positives by the classifier consistently in the 200 instances (across every sample in every run). **Fig. S5:** Heatmap of recurrent false negative families. This heatmap displays the family level classification that were identified as false negatives by the classifier between 50 to 200 on 200 instances. **Fig. S6:** Taxonomic comparison across sampling sites and soil horizons at phylum level. (a) Bar plot detailing the microbial composition from the shotgun dataset using Kraken2 (custom), displaying the older phylum names. (b) Bar plot showcasing the microbial composition as presented in the original study. The top 14 phyla are represented with distinct colours in both plots, while all other phyla are grouped as “Remainder”. A consistent colour palette is maintained across both plots for equivalent phyla. The x-axis illustrates the three distinct sampling sites (Podsol, Cambisol, and Stagnosol), further divided by soil horizons (O, A, and B) within each site. Note: This supplementary figure provides a reference for older phylum nomenclature in comparison to the main text. **Fig. S7:** Impact of different classifiers on beta diversity analysis of microbial communities across soil samples. The analysis reveals a significant influence of the chosen classifier on the distribution of microbial communities. Presented are the Bray-Curtis dissimilarity plots at (a) species level (PerMANOVA: 0.85, p<0.001) and (b) genus level (PerMANOVA: 0.78, p < 0.001). 

**Additional file 2. Table S1:** The R packages, version and tools utilised in this study. **Table S2:** Comparative mpact of tools and abundance thresholds on F1 Measure: Results from the Dunn Test. Key outcomes from the post-hoc Dunn test underscore the impact of various tools and relative abundance threshold on the F1 measure. Here, Z values represent the standard Z scores, and any p-value below 0.05 was deemed statistically significant. **Table S3a:** Kruskal-Wallis analysis examining the influence of tools and thresholds on Euclidean distance per sample. Data was grouped by 'Run' and 'Classifier'. Each row indicates differences in Euclidean distance for specific groupings. The H-values, derived from the Kruskal-Wallis test statistic, consistently indicate significant differences between groups. Effect sizes are denoted by the eta2[H] values, which fall within an approximate range of 0.902 to 0.950. All associated p-values are less than 0.0001. **Table S3b:** Significant findings from the post-hoc Dunn test: This table highlights the effects of different tools and threshold values on the Euclidean distance, calculated for each sample in every run. **Table S4:** Chi-squared Analysis of Classification Outcomes by Different Tools at Family Level, Showing All Outcomes (FN, FP, TN, TP) with Significant P-values.

**Additional file 3.** Species level taxonomic classification of in-silico data: Preliminary, unfiltered results without any relative abundance threshold applied.

**Additional file 4.** Genus level taxonomic classification of in-silico data: Preliminary, unfiltered results without any relative abundance threshold applied.

**Additional file 5.** Family level taxonomic classification of in-silico data: Preliminary, unfiltered results without any relative abundance threshold applied.

**Additional file 6.** Species level taxonomic classification results from assembled contigs, in-silico data: Preliminary, unfiltered results without any relative abundance threshold applied.

**Additional file 7.** Metadata file for in-silico data.

**Additional file 8.** Metadata file for real dataset.

**Additional file 9.** Results of phylum-level taxonomic classification from the real dataset using Kraken2 (custom). Relative abundance threshold: not applied.

**Additional file 10.** Results of species-level taxonomic classification from the real dataset. Relative abundance thresholds applied are 0.001% for Kraken2 (custom) and Kaiju, and 0.005% for Kraken2 (default).

**Additional file 11.** Results of genus-level taxonomic classification from the real dataset. Relative abundance thresholds applied are 0.001% for Kraken2 (custom) and Kaiju, and 0.005% for Kraken2 (default).

**Additional file 12.** SoilGenomeDB: Comprehensive metadata of soil-specific bacterial, archaeal, and fungal genomes from NCBI.

## Data Availability

The scripts employed for sequencing data simulation, bioinformatics data processing, and taxonomic classification can be found at https://github.com/roseedwin/Benchmarking-taxonomic-classifiers-for-soil-shotgun-data. The environmental sequencing data were downloaded from the NCBI Sequence Read Archive (SRA) and can be accessed using BioProject identifiers PRJNA717813 and PRJNA717918. The taxonomic classification results and metadata files that support the study along with the in-silico soil mock community metadata generated have been included as Additional file 3, 4, 5, 6, 7, 8, 9, 10, 11, 12.

## Abbreviations

CPU: Central processing unit
FP: False positive
FN: False negative
GTDB: Genome Database Taxonomy
MAG: Metagenome Assembled Genome
MDS: Multidimensional scaling
NCBI: National Center for Biotechnology Information
PERMANOVA: Permutational multivariate ANOVA
SRA: Sequence read archive
TP: True positive
TN: True negative
