# Supplementary material for: An in-depth evaluation of metagenomic classifiers for soil microbiomes
Source: Environ Microbiome. 2024 Mar 28;19:19. doi: 10.1186/s40793-024-00561-w (PMC10979606; doi:10.1186/s40793-024-00561-w)

# SUPPLEMENTARY FIGURES:

# Supplementary figure 1. Venn diagrams depicting classifier disparities at the species level for false positives (a), false negatives (b), and true positives (c). Differential true positives at family level are shown in (d).


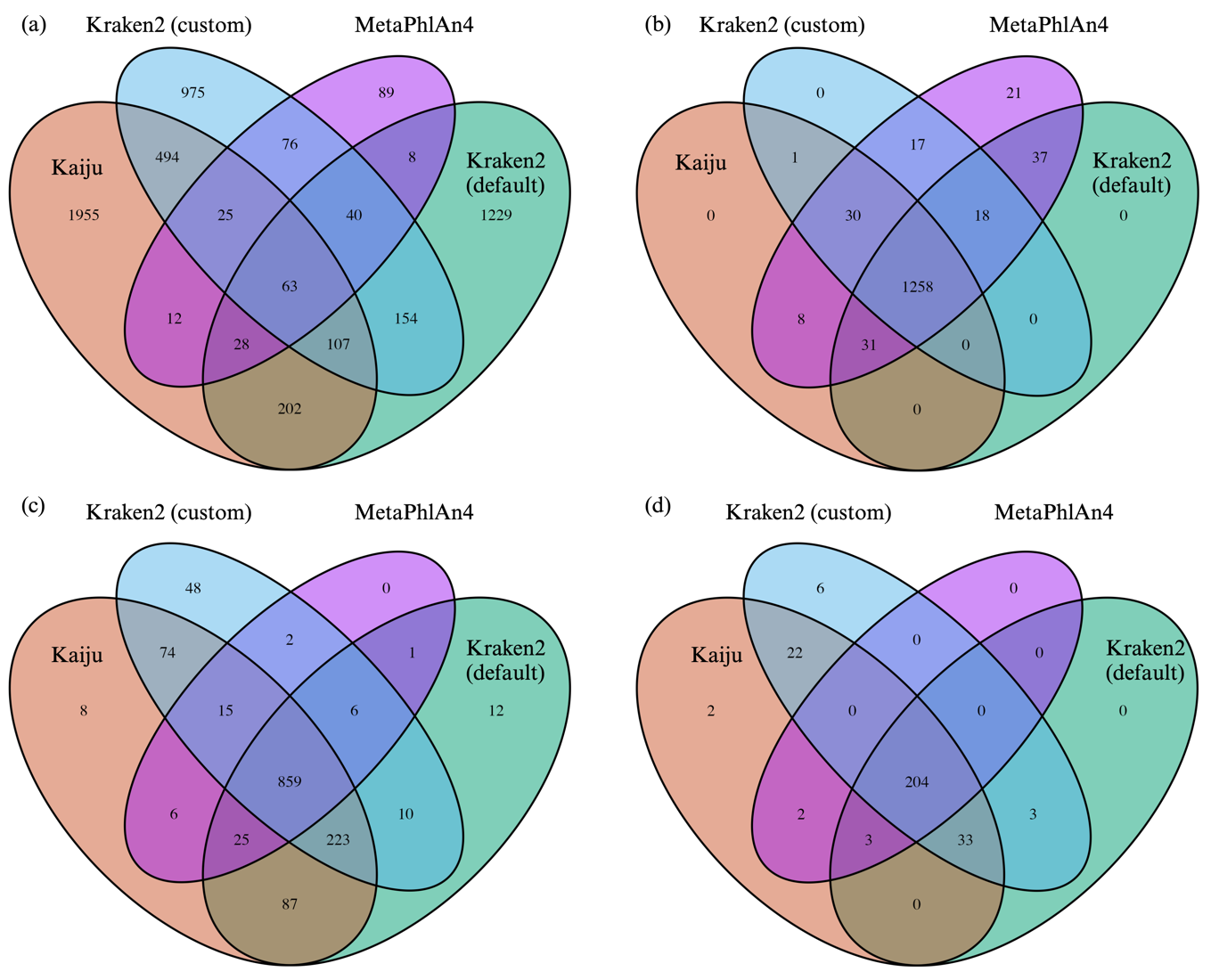


#### Supplementary figure 2. Comparison of F1 score, sensitivity and precision for different taxonomic classifiers on assembled reads and QC/ trimmed reads.


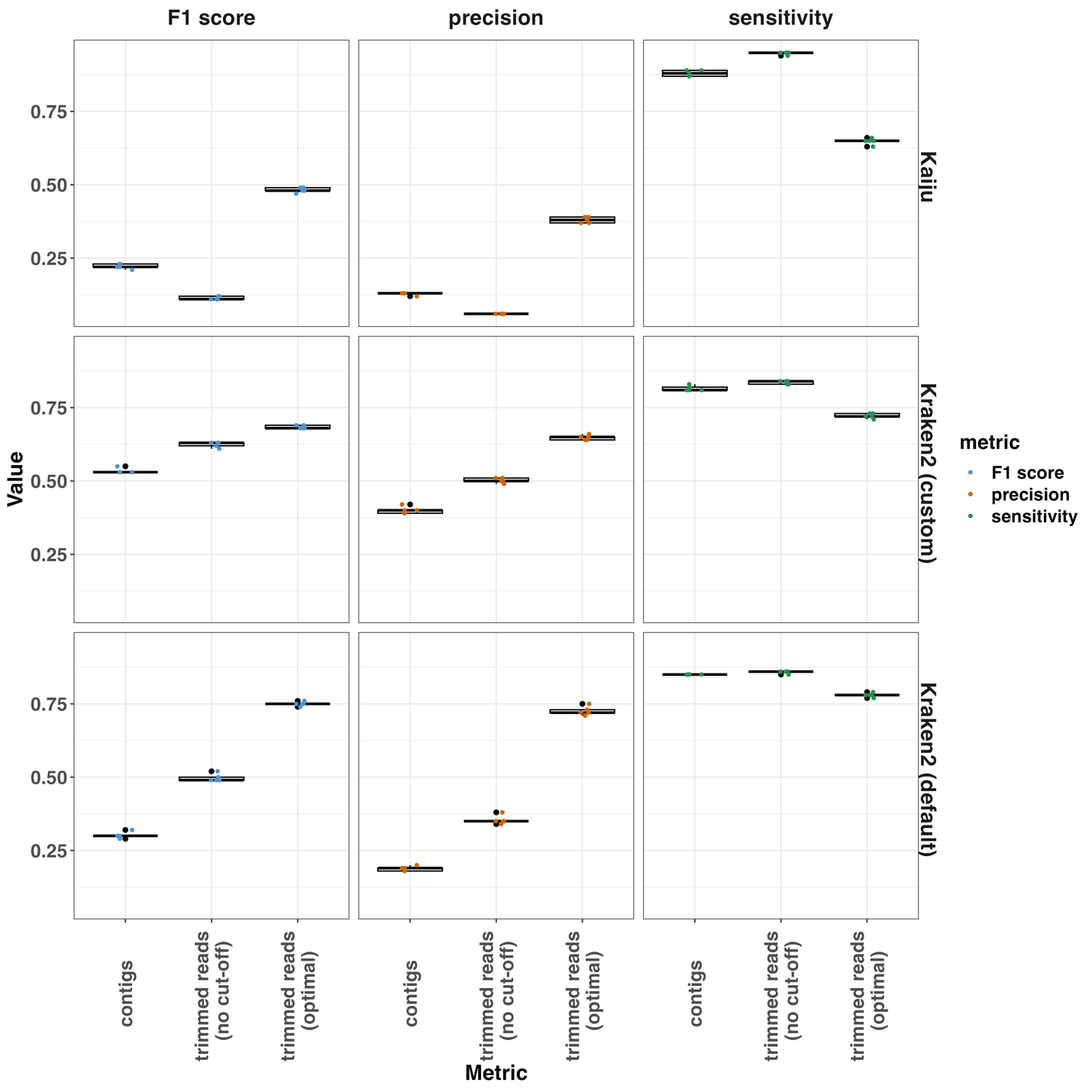


#### Supplementary figure 3. Balloon Plot Visualization of Classification Outcomes at the Family Level Using Different Tools. The size of each balloon corresponds to the count of observations under specific outcome categories: FN (False Negatives), FP (False Positives), TN (True Negatives), and TP (True Positives). The colour gradient within the balloons indicates the magnitude and direction of deviations between observed and expected classifications. Numbers displayed on the right or bottom of the plot represent cumulative counts across respective categories.


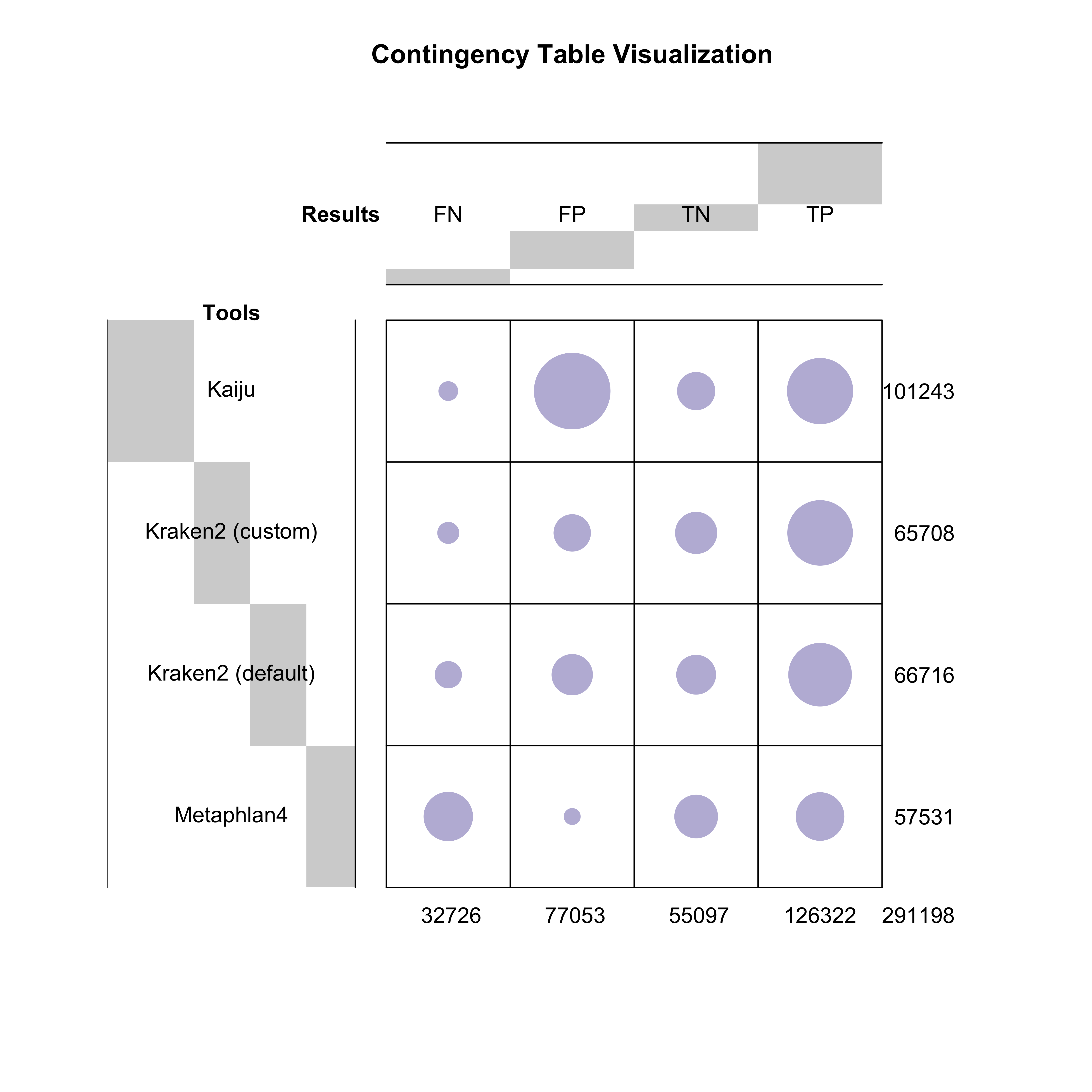


#### Supplementary figure 4. Heatmap representing consistently misclassified families. This heatmap displays the family level classification that were identified as false positives by the classifier consistently in the 200 instances (across every sample in every run)

**
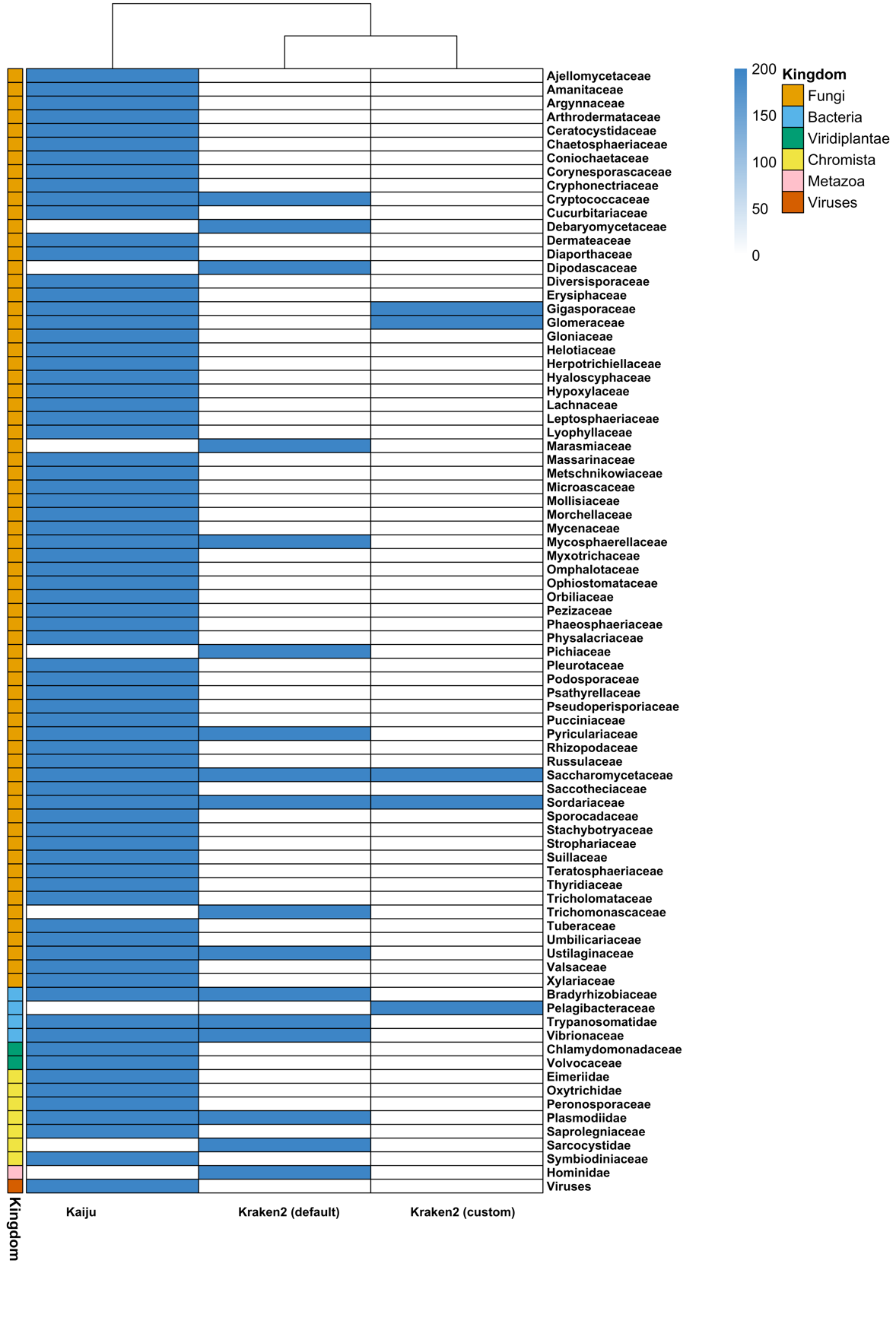
**

#### Supplementary figure 5. Heatmap of recurrent false negative families. This heatmap displays the family level classification that were identified as false negatives by the classifier between 50 to 200 on 200 instances.

**
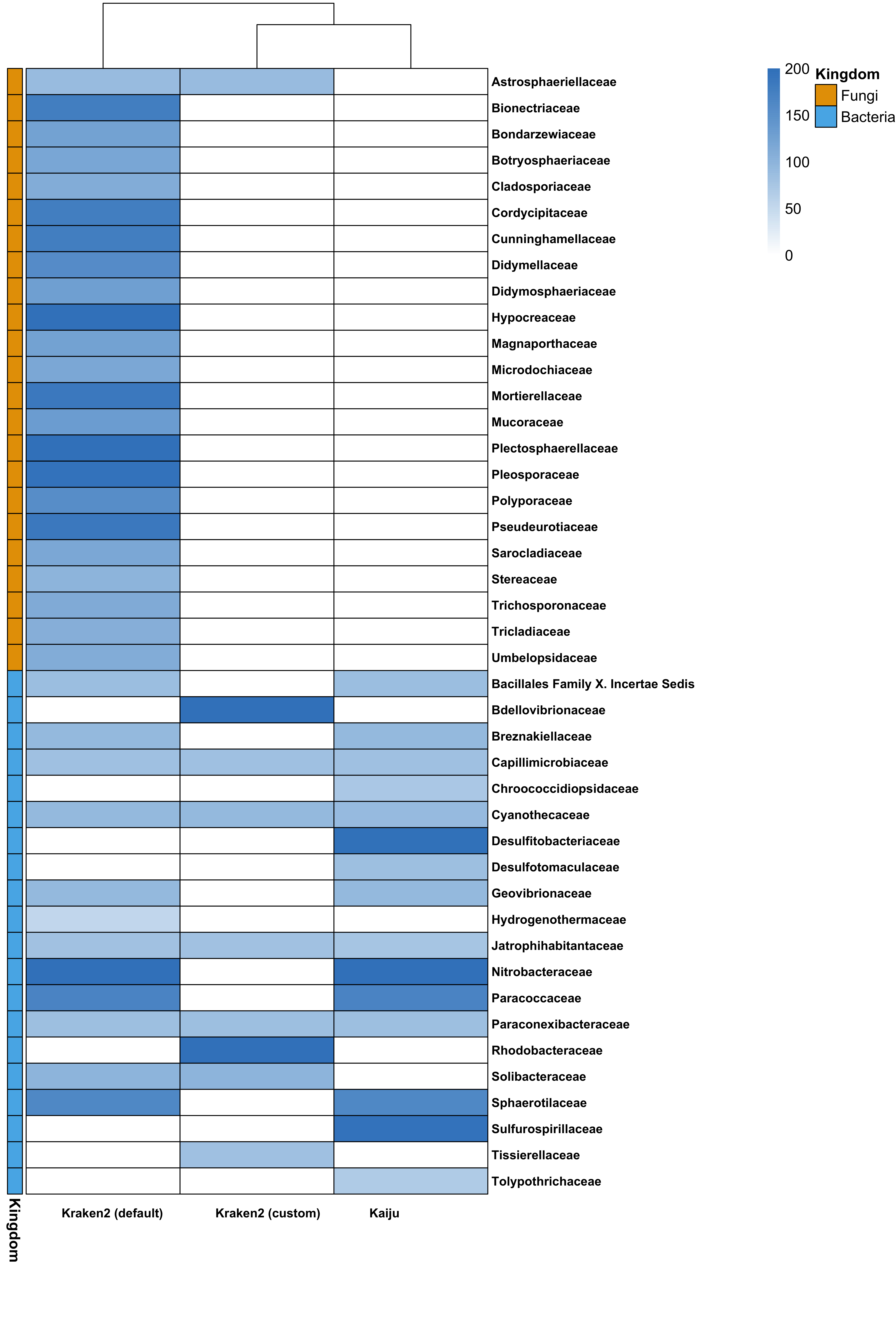
**

#### **Supplementary figure 6.** Taxonomic comparison across Sampling Sites and Soil Horizons at phylum level. (a) Bar plot detailing the microbial composition from the shotgun dataset using Kraken2 (custom), displaying the older phylum names. (b) Bar plot showcasing the microbial composition as presented in the original study. The top 14 phyla are represented with distinct colours in both plots, while all other phyla are grouped as “Remainder”. A consistent colour palette is maintained across both plots for equivalent phyla. The x-axis illustrates the three distinct sampling sites (Podsol, Cambisol, and Stagnosol), further divided by soil horizons (O, A, and B) within each site. Note: This supplementary figure provides a reference for older phylum nomenclature in comparison to the main text.

**
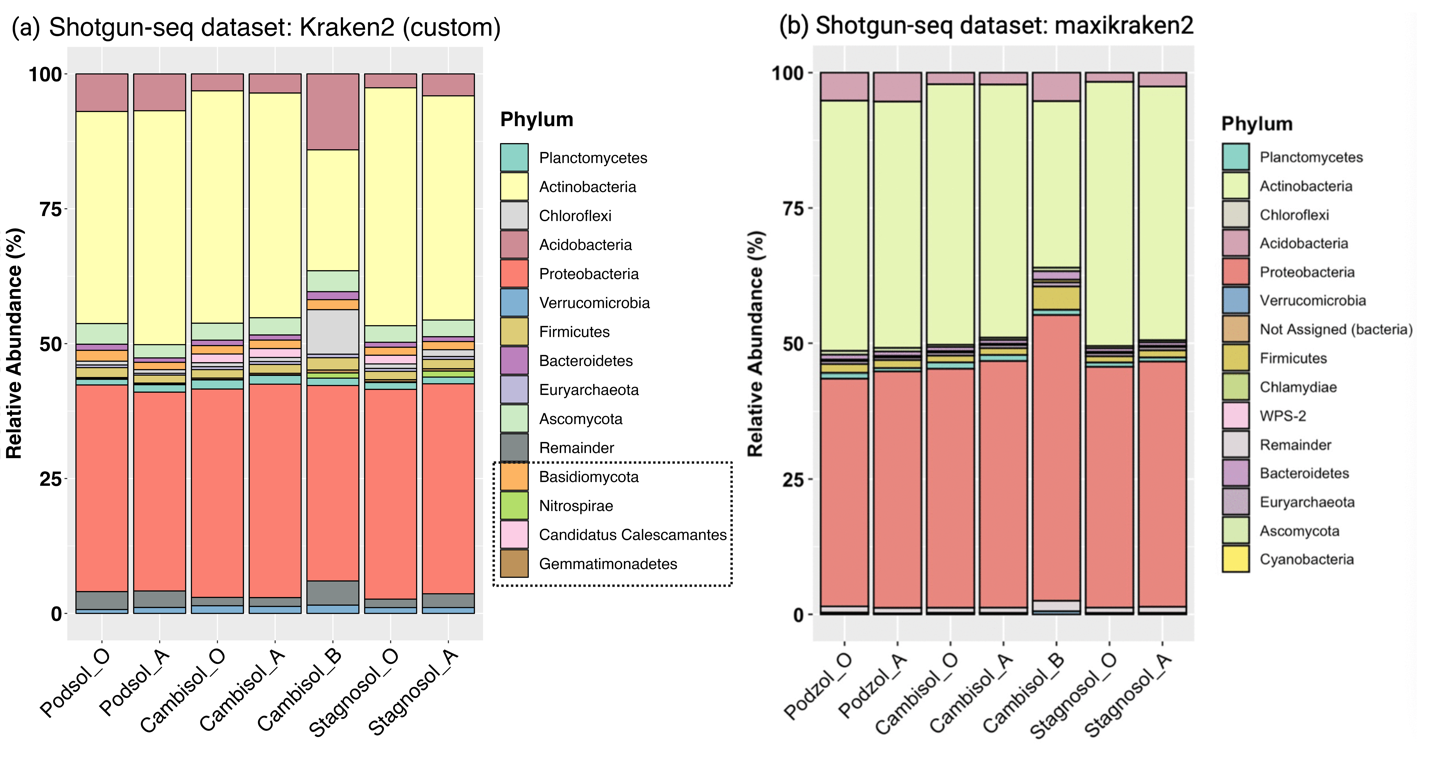
**

**Supplementary figure 7.** Impact of different classifiers on beta diversity analysis of microbial communities across soil samples. The analysis reveals a significant influence of the chosen classifier on the distribution of microbial communities. Presented are the Bray-Curtis dissimilarity plots at (a) species level (PerMANOVA: 0.85, p < 0.001) and (b) genus level (PerMANOVA: 0.78, p < 0.001).


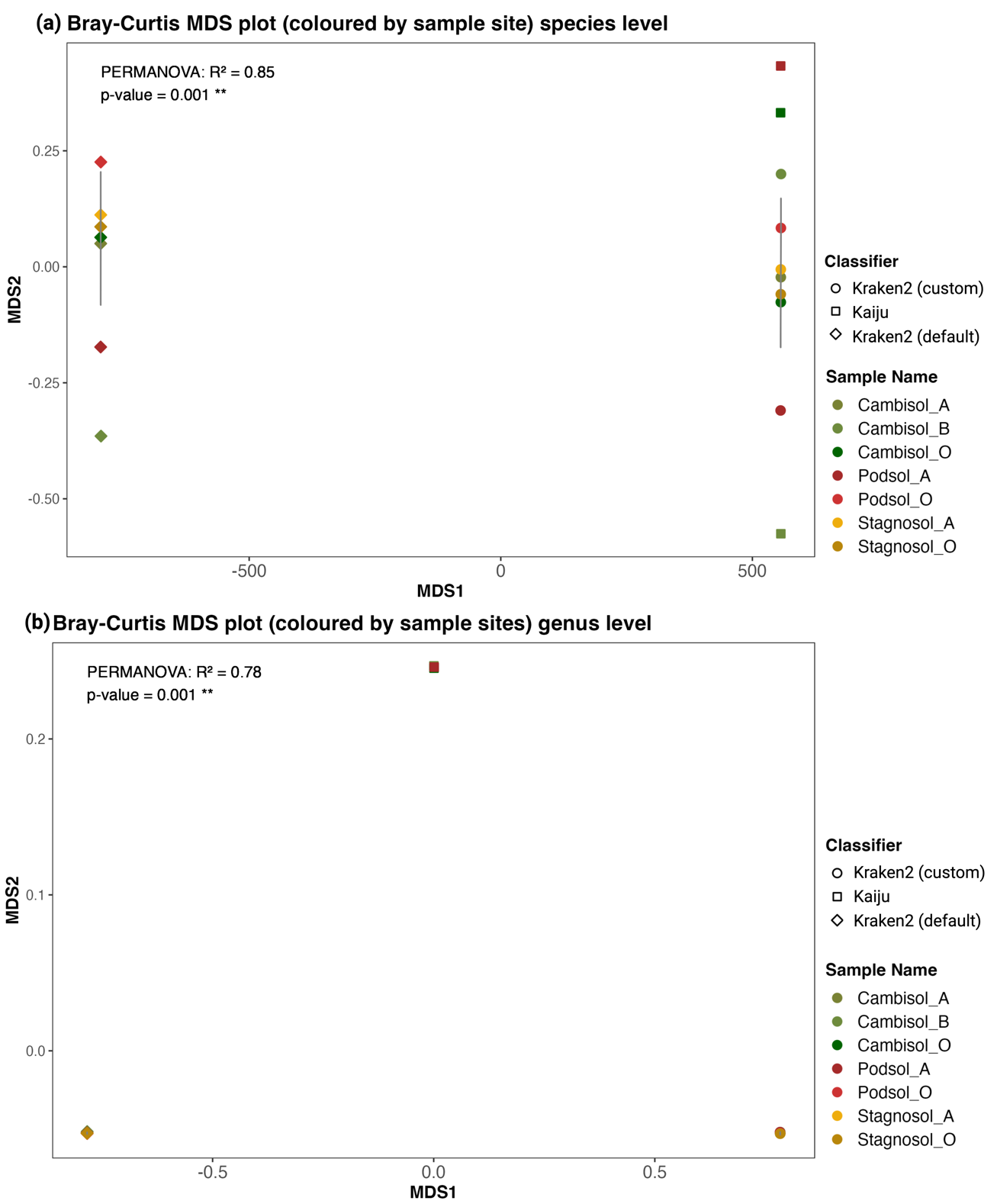

Supplement: Supplementary file 1 — Additional file 1. Fig S1: Venn diagrams depicting classifier disparities at the species level for false positives (a), false negatives (b), and true positives (c). Differential true positives at family level are shown in (d). Fig. S2: Comparison of F1 score, sensitivity and precision for different taxonomic classifiers on assembled reads and QC/ trimmed reads. Fig. S3: Balloon plot visualization of classification outcomes at the family level using different tools. The size of each balloon corresponds to the count of observations under specific outcome categories: FN (False Negatives), FP (False Positives), TN (True Negatives), and TP (True Positives). The colour gradient within the balloons indicates the magnitude and direction of deviations between observed and expected classifications. Numbers displayed on the right or bottom of the plot represent cumulative counts across respective categories. Fig. S4: Heatmap representing consistently misclassified families. This heatmap displays the family level classification that were identified as false positives by the classifier consistently in the 200 instances (across every sample in every run). Fig. S5: Heatmap of recurrent false negative families. This heatmap displays the family level classification that were identified as false negatives by the classifier between 50 to 200 on 200 instances. Fig. S6: Taxonomic comparison across sampling sites and soil horizons at phylum level. (a) Bar plot detailing the microbial composition from the shotgun dataset using Kraken2 (custom), displaying the older phylum names. (b) Bar plot showcasing the microbial composition as presented in the original study. The top 14 phyla are represented with distinct colours in both plots, while all other phyla are grouped as “Remainder”. A consistent colour palette is maintained across both plots for equivalent phyla. The x-axis illustrates the three distinct sampling sites (Podsol, Cambisol, and Stagnosol), further divided by soil horizons (O, [file 40793_2024_561_MOESM1_ESM.docx]
